# Photocatalytic Depolymerization of Lignin: C-O Bond Cleavage in β-O-4 Models Using S-Doped Ultra-Thin Bi_3_O_4_Cl Nanosheets

**DOI:** 10.3390/molecules29245979

**Published:** 2024-12-18

**Authors:** Chunli Jiang, Sixue Zhang

**Affiliations:** 1 School of Chemistry, Sun Yat-Sen University, Guangzhou 510275, China; 2 School of Chemistry and Chemical Engineering, Guangxi Minzu University, Nanning 530006, China; z18324553622@163.com; 3 Hui Zhou Research Institute, Sun Yat-Sen University, Huizhou 516000, China

**Keywords:** S-doped Bi_3_O_4_Cl, β-O-4 lignin model, C-O bond cleavage, interface engineering

## Abstract

The selective depolymerization of β-O-4 lignin models into high-value aromatic monomers using photocatalysis presents both significant opportunities and challenges. Photocatalysts often face issues such as high photogenerated carrier recombination rates and limited operational lifetimes. This study introduces S doping to modulate the surface interface of Bi_3_O_4_Cl (BOC) nanosheets, enhancing C-O bond cleavage efficiency in β-O-4 lignin models under visible light at ambient temperatures. Comprehensive characterization, including atomic force microscopy (AFM), transmission electron microscopy (TEM), X-ray photoelectron spectroscopy (XPS), electron spin resonance (ESR), and density functional theory (DFT) analysis, revealed that S doping reduces BOC nanosheet thickness to 1.51 nm and promotes charge carrier separation, thereby generating greater concentrations of reactive species, specifically •O_2_^−^ and •OH. Photocatalytic depolymerization experiments demonstrated that S-doped BOC achieved a C-O bond cleavage selectivity of 93% and an aromatic monomer yield of 629.03 μmol/g/h (i.e., 1.5 times higher than that of undoped BOC). This work provides a strategic approach to designing photocatalysts with enhanced selectivity and efficiency for lignin depolymerization.

## 1. Introduction

In the future, the comprehensive utilization of biomass resources is anticipated to supplant fossil fuels in the production of high-value aromatic compounds. Lignin, a significant non-carbohydrate component of biomass, has been extensively studied and employed in the synthesis of aromatic chemicals [1]. It is characterized as an aromatic heterogeneous biopolymer with a three-dimensional, cross-linked network structure, constituting approximately 15–30 wt.% of lignocellulosic biomass [2]. Lignin is composed of three primary monomers: *p*-hydroxyphenyl (H), guaiacyl (G), and syringyl (S) [3,4,5] units. The predominant inter-monomeric bonds are formed by β-O-4 linkage, which, due to their labile nature, are critical for the depolymerization of lignin [6,7]. However, conventional methods of lignin depolymerization often necessitate harsh reaction conditions, which are frequently accompanied by undesirable by-products. Therefore, it is essential to explore efficient and environmentally friendly strategies for lignin depolymerization.

In recent years, semiconductor-based photocatalysis has garnered significant attention due to its advantages, including mild reaction conditions, simple reaction steps, low costs, and the absence of the need for further purification. Consequently, it is considered a potential alternative to traditional technologies. During the photocatalytic process, photogenerated carriers and species can effectively cleave target bonds under simulated sunlight, resulting in the generation of aromatic monomers. Currently, there are two primary methods for C-O bond cleavage: the two-step cleavage method, which involves breaking the bond following the pretreatment of lignin, and the direct cleavage method [8,9]. Stephenson [10] utilized Ir to pretreat a lignin model, obtained ketones and β-hydroxyketones as depolymerization products, and subsequently employed Pb(OAc)_2_ to catalyze the cleavage of C_β_-O bond, yielding phenol and acid under blue light. Wang [11] replaced the aforementioned two-step reaction with a one-pot method, wherein Pd/ZnIn_2_S_4_ oxidized the alcohol lignin model at 455 nm, and ketones were obtained from the mentioned depolymerization reactions, followed by depolymerization at 365 nm using TiO_2_. The selectivity for monomeric acids and phenols achieved through the oxidation-hydrolysis series method exceeded 90%. Therefore, there is a pressing need to explore catalysts that exhibit a high response to visible light.

The authors’ previous study of the typical photocatalytic redox process [12], the photocatalytic cleavage of β-O-4 lignin model using CuO/BiVO_4_ at room temperature, indicates that the presence of superoxide radicals •O_2_^−^ significantly enhances the photocatalytic reaction. Consequently, it is necessary to identify an effective catalyst for O_2_ adsorption and reduction. Bismuth halide oxides (BiOX) are of particular interest due to their unique crystal structures, excellent electronic properties, low toxicity, and ease of synthesis [13]. Presently, most research has concentrated on BiOCl. However, the unique structure of BiOCl results in a limited response to visible light, thereby constraining its practical applications [14]. Bi_3_O_4_Cl (BOC), a variant of BiOX, shares a similar layered structure with BiOCl, but its interlayer configuration consists of [Bi_3_O_4_] units interspersed with two layers of [Cl] ions [15]. In comparison to BiOCl, BOC possesses a narrower band gap, which enhances its responsiveness to visible light. Despite this increased responsiveness, the narrow band gap also contributes to a rapid recombination rate of photogenerated carriers, leading to a suboptimal photocatalytic effect. To address this issue, this study focused on modifying the electronic structure of BOC nanosheets to improve their visible light absorption and, consequently, their photocatalytic activity [13]. Common strategies for such modifications include doping, introducing defects, forming heterojunctions, controlling crystal planes and morphology, and depositing precious metals. Among these, nonmetal doping is regarded as an effective and feasible modification technique, as it can create defects on the catalyst surface and increase the sites available for O_2_ adsorption during the doping process [16]. It is also known that in the solar spectrum, UV light does not use more than 5%, visible light uses less than 45%, and near-infrared (NIR) light uses approximately 50% of the solar light energy. Thus, in view of efficient solar energy utilization, efforts need to be made to extend the light response from UV to visible light. To date, there have been no reports on the adjustment of the visible light absorption range of BOC nanosheets through nonmetal doping.

Through an investigation of the properties of various nonmetallic dopants, it is determined that S exhibits high thermal stability and has the potential to enhance the absorption of visible light. Therefore, this study involved the modification of the catalyst through S doping, utilizing a simple hydrothermal method. A series of single-factor experiments revealed that the depolymerization of lignin models demonstrated exceptional photocatalytic performance when the S doping concentration was set at two wt.%. Under simulated sunlight conditions over a duration of 10 h, a yield of 99% was achieved by cleaving the C_β_-O bond of β-O-4 lignin models. These results underscore the potential of photocatalysis in the depolymerization of lignin, facilitating the production of value-added aromatic commercial chemicals and representing a significant advancement toward the mild and selective processing of lignin.

## 2. Results and Discussion

**Figure 1:**
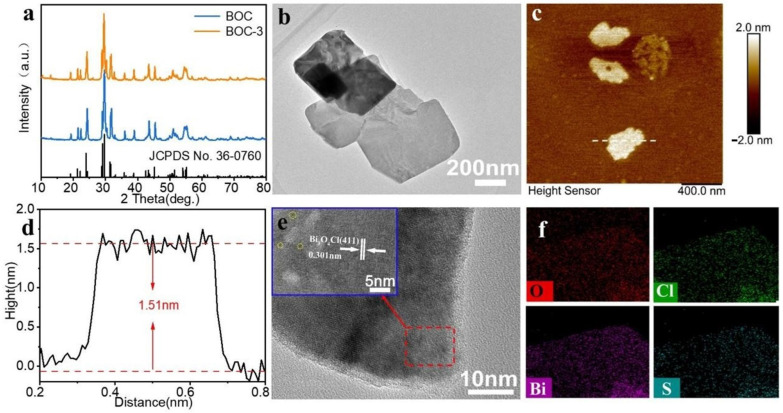
Structural and morphological characterization of S-doped BOC-3 nanosheets: (**a**) XRD patterns of undoped BOC and S-doped BOC-3 samples; (**b**) TEM images of BOC-3; (**c**) AFM images and (**d**) the corresponding height profiles of BOC-3; (**e**) HRTEM image of BOC-3; (**f**) EDS mapping of BOC-3.

S-doped ultrathin BOC nanosheets were synthesized using a simplified secondary hydrothermal method (Figure S1a). The crystalline phases of BOC and BOC-3 were analyzed through XRD (Figure 1a). All diffraction angles were indexed to monoclinic BOC (JCPDS No. 36-0760) [17,18] with no additional diffraction angles detected, indicating that BOC possesses a single monoclinic structure. Notably, as the amount of S doping increased, the intensity of all peaks slightly diminished without the emergence of any impurities, suggesting that S doping did not alter the crystal structure of BOC. The morphological characteristics of the synthesized ultrathin BOC and BOC-3 nanosheets were further examined using scanning electron microscopy (SEM), while the effects of S doping on their structure were verified through AFM and TEM. SEM images of BOC and BOC-3 nanosheets are illustrated in Figure S1b,c. BOC nanosheets exhibit an irregular sheet-like morphology, and S doping did not significantly impact this morphology. Furthermore, AFM images of BOC and BOC-3 (Figure S1d and Figure 1c), along with their corresponding height profiles (Figure S1e and Figure 1d), indicate that the thicknesses of the as-prepared BOC and BOC-3 are approximately 4.97 nm and 1.51 nm, respectively. The undoped BOC corresponds to the thickness of five monolayers of BOC plates, suggesting that the secondary hydrothermal doping may have led to the interlayer stripping of BOC. In the case of the BOC-3 composite, nanosheets with transverse sizes ranging from 200 nm to 300 nm were observed. Additionally, HRTEM images (Figure 1b,e) of BOC-3 nanosheets reveal different lattice fringes, with a lattice fringe spacing of 0.301 nm corresponding to the (411) plane of BOC. Irregular regions are visible in the circled areas of BOC-3, indicating the formation of clustered-point defects in the selected region. Additionally, energy dispersive spectroscopy (EDS) mapping demonstrates that S is uniformly doped on the surface of BOC nanosheets, as illustrated by the color intensities in Figure 1f. This result further corroborates that S-element doping modulates the surface characteristics of BOC nanosheets.

The molecular and layered structures of BOC and BOC-3 were analyzed using Raman spectroscopy, with the corresponding spectra in Figure S2. The peaks observed at 83 cm^−1^, 96 cm^−1^, 145 cm^−1^, 205 cm^−1^, 389 cm^−1^, and 633 cm^−1^ are attributed to BOC. Specifically, the peaks at 80 cm^−1^, 93 cm^−1^, and 145 cm^−1^ correspond to A_1_g external and internal Bi-Cl stretching modes of BOC, respectively [19]. The peak at 205 cm^−1^ is associated with Eg internal stretching of BOC, while the peak at 395 cm^−1^ is attributed to the vibrations of Eg and B1 modes resulting from the positional shift of the O atom within the crystal lattice. Notably, the incorporation of S into the lattice leads to a reduction in the intensity of Raman diffraction peaks, as well as a broadening and shift to lower energy. This observation indicates the successful doping of the S element into the BOC lattice. The resultant quantum domain effects arising from the reduction in material thickness, along with phonon-limited domains due to defects, contribute to the observed shifts in peak positions. In summary, XRD and Raman analyses provide sufficient evidence to confirm the successful synthesis of BOC-3 through the substitution of Cl atoms.

**Figure 2:**
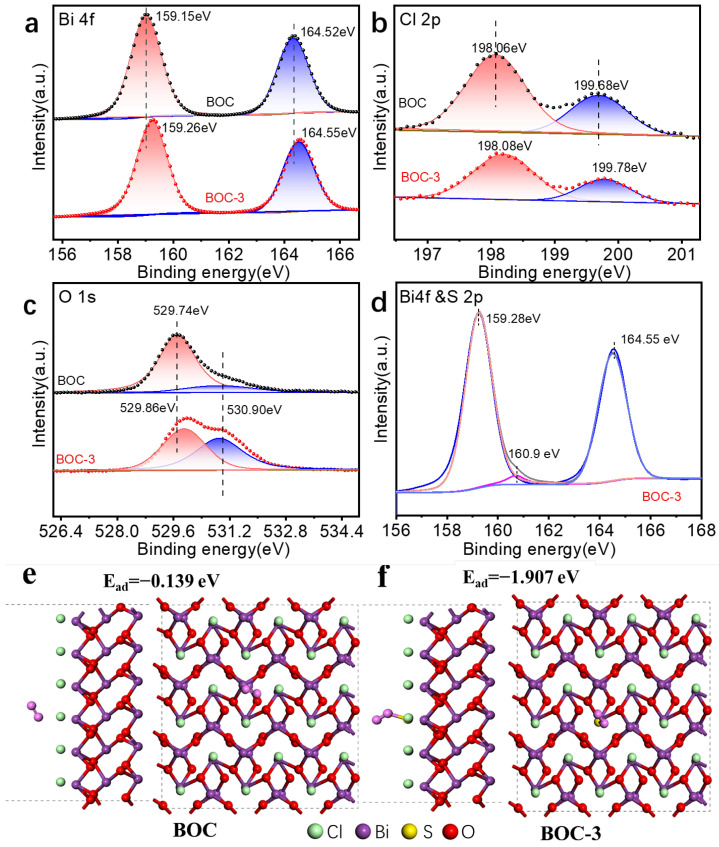
High-resolution XPS spectra and O adsorption simulation of S-doped BOC-3: (**a**) Bi 4f, (**b**) Cl 2p, (**c**) O 1s, and (**d**) S 2p; DFT-based simulation of O_2_ adsorption energy on (**e**) BOC and (**f**) BOC-3 hybrid.

The chemical states and surface composition of BOC and BOC-3 samples were further examined using XPS. In Figure S3, an XPS survey spectrum revealed the presence of the expected elements (e.g., Bi, O, Cl, and S) in the BOC-3 nanosheet, which aligns with the results obtained from EDS mapping. The Bi 4f spectrum (Figure 2a) exhibited binding energies of 159.15 eV (4f_7/2_) and 164.52 eV (4f_5/2_), corresponding to Bi^3+^ [20]. In Figure 2b, Cl 2p spin-orbit levels of both BOC and BOC-3 split into two peaks at 198.06 eV and 199.68 eV, which are attributed to Cl 2p_3/2_ and 2p_1/2_ orbitals of Cl^−^, respectively [18]. Notably, the peak positions did not change significantly after S doping, indicating that the incorporation of S did not substantially impact the interlayer Cl^−^ [21]. In Figure 2c, the S 2p spectrum displays a single peak at 160.9 eV, suggesting the presence of S^2−^ [17,22]. In the high-resolution O 1s XPS spectra (Figure 2d), two fitting peaks at 529.2 eV and 530.9 eV were identified, which are attributed to Bi^3+^.

O bonds and $V_{Ö}$ [17]. The intensity of O vacancies in S-doped BOC was significantly greater than that in the undoped BOC, indicating that doping facilitates the detangling of O atoms within the catalyst lattice, resulting in O_2_ deficiency and the formation of defects [23]. This observation is consistent with the results obtained from HRTEM analysis. To further investigate the relationship between doping and O_2_ adsorption, DFT was employed to analyze the adsorption of O_2_ on the catalyst surface. In Figure 2e,f, the adsorption energy of BOC-3 for O is lower than that of BOC, suggesting that S doping enhances O adsorption on the catalyst surface. Collectively, these results confirm the successful synthesis of BOC-3 and demonstrate that S doping can improve the adsorption and O activation on the surface of BOC-3.

**Figure 3:**
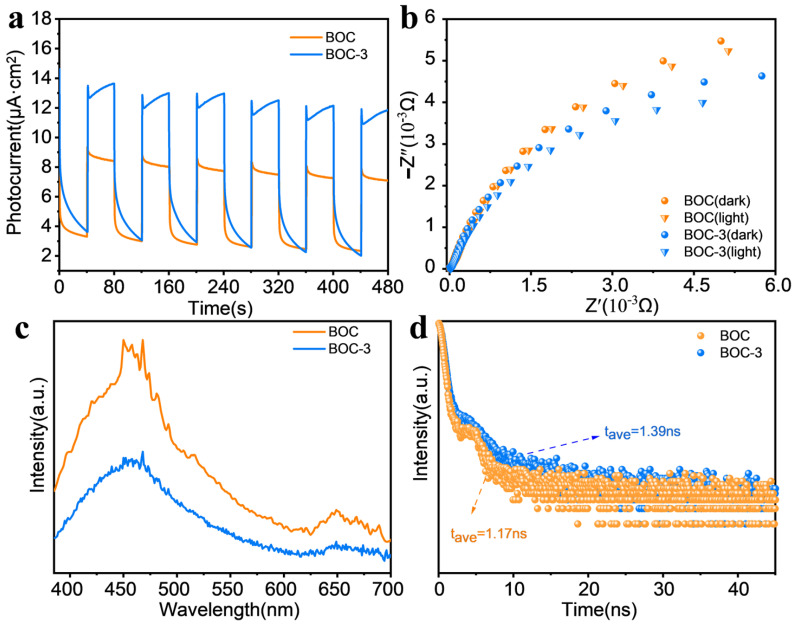
Photophysical and electrochemical characterization of S-doped BOC-3 for photocatalytic performance: (**a**) time-dependent photocurrent responses of BOC and BOC-3; (**b**) Nyquist plots of BOC and BOC-3 in the dark and under illumination; (**c**) steady-state PL spectra (excitation at 365 nm); and (**d**) time-resolved PL decay plots registered at 450 nm of BOC and BOC-3.

Existing studies suggested that the recombination rate of photocarriers significantly impacted the performance of the catalyst [24]. Therefore, transient photocurrent densities, EIS, and PL were employed to investigate charge separation and transport mechanisms. It is worth noting that each vacancy is characterized by a double-positive charge, therefore being a trap for photelectrons. Photocurrent responses were observed upon the excitation of BOC and BOC-3 under visible light irradiation (Figure 3a). The results indicated that BOC-3 exhibited a markedly enhanced photocurrent density, suggesting that S doping inhibits the recombination of electrons e^−^-h^+^. Furthermore, the arc radius of BOC-3 in Nyquist plots of EIS is smaller than that of pristine BOC (Figure 3b), indicating that the charge transfer resistance is reduced following S doping. This reduction promotes the transfer of photogenerated carriers to the surface reactivity center, thereby participating in the catalytic reaction [25]. These results show that the defect state reduces the work function, resulting in electrons being more easily transferred to the semiconductor surface to participate in chemical reactions. The separation of photogenerated carriers was detected by assessing their photoluminescence (PL) spectrum (Figure 3c) [13]. The PL intensity of BOC-3 was lower than that of BOC, which supports the notion that S doping enhances the transport of photocarriers while inhibiting the recombination of electrons and holes. Additionally, a TRPL emission spectrum was recorded for the S-doped structures to investigate the dynamics of charge carriers. Compared to BOC (1.17 ns), BOC-3 exhibits a slightly longer average emission lifetime (1.39 ns), as depicted in Figure 3d. This suggests that BOC-3 is more effective in separating electron-hole pairs than BOC when photogenerated electrons and holes are present.

The photocatalyst absorbs sunlight and converts it into chemical energy, representing the initial step of the reaction. Consequently, the optical absorption characteristics and band structure of BOC and BOC-3 catalysts were analyzed using UV-vis DRS and Mott–Schottky plots. In Figure S4a, the absorption edge of BOC is observed at 439 nm, whereas BOC-3 exhibits a shift to 491 nm. This significant red shift indicates that S doping enhances the absorption of visible light and affects the band gap of the catalyst [22]. The band gaps of BOC and BOC-3 were calculated to be 2.86 eV and 2.64 eV, respectively, using a Kubelka–Munk plot (Figure S4a). Mott–Schottky measurements were conducted to further confirm the type and band potential of the photocatalysts. In Figure S4b, both materials display positive slopes, indicating that the catalysts are typical N-type semiconductors [26]. The flat band potentials were determined to be −0.12 V and −0.43 V (vs. Ag/AgCl) for BOC and BOC-3, respectively. According to the empirical equation E_VB_ = E_CB_ + Eg [27], the valence band (VB) potentials of BOC and BOC-3 are estimated to be 2.64 eV and 2.11 eV, respectively. Based on this research, the energy band diagrams for BOC and BOC-3 were developed (Figure S7). On the one hand, the doping process can generate impurity levels within the interlayer of the BOC, thereby promoting the separation and migration of photogenerated charge carriers [28]. On the other hand, the introduction of impurities may create defects on the surface of the BOC, which can enhance the adsorption of O_2_ on the catalyst surface and further improve reaction efficiency. These results are consistent with electrochemical analyses and DFT calculations.

**Figure 4:**
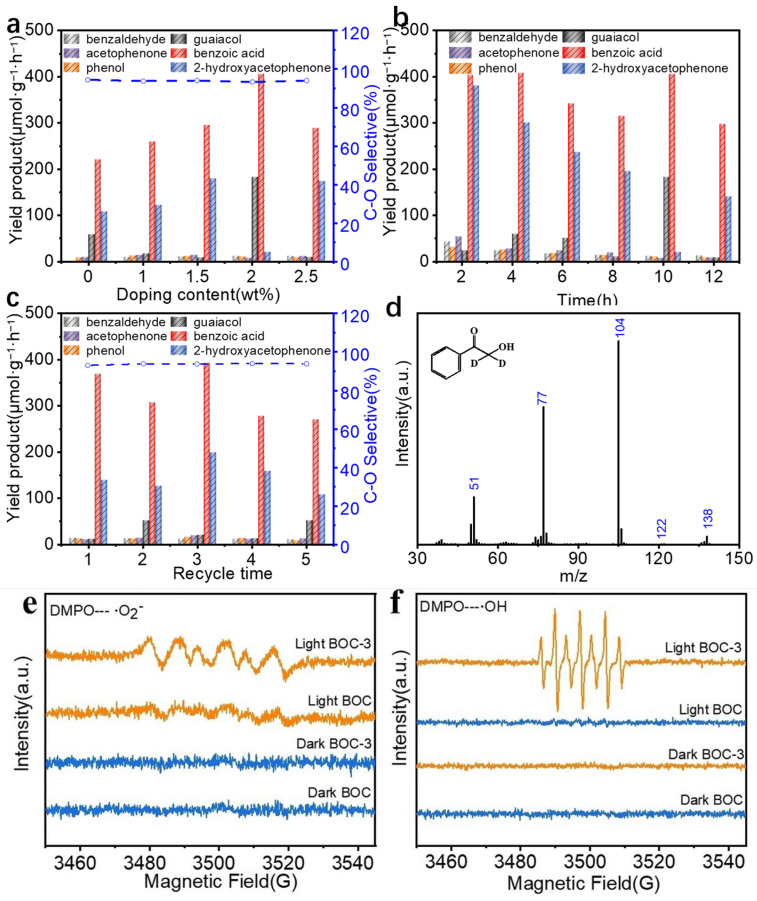
Photocatalytic efficiency, stability, and reactive species analysis of S-Doped BOC-3 in lignin model depolymerization: (**a**) comparative photocatalytic C-O bond cleavage performance of BOC-3 and control samples on lignin β-O-4 model; (**b**) products yield as a function of reaction time; (**c**) stability test of BOC-3; (**d**) GC-MS analysis of phCOCD_2_OH; (**e**) ESR spectra showing O_2_^−^; and (**f**) •OH in BOC and BOC-3 under visible light.

The β-O-4 bond is of significant importance for the production of low-molecular-weight aromatic compounds. In this study, 2-(2-methoxy-phenoxy)-1-acetophenone was selected as a model compound for lignin. The study investigated the effect of varying S loadings on the photocatalytic degradation of the lignin model. In Figure 4a, the effect of different S loading levels on the photocatalytic cleavage of the C-O bond in the lignin model was assessed. The BOC-3 catalyst demonstrated a notably high photocatalytic efficiency, achieving a conversion rate of 99.4% and a C-O cleavage selectivity of 93.3%. This enhancement increased with the amount of S doping before gradually declining. This observation suggests that moderate $V_{Ö}$ on the catalyst’s surface can enhance the separation efficiency of photo-induced carriers, while excessive $V_{Ö}$ may act as recombination centers, which are detrimental to the separation and transfer of photo-induced carriers [29,30]. Therefore, BOC-3 was selected for further photocatalytic testing. The effect of reaction time on product formation is illustrated in Figure 4b, which shows that the accumulation of products increased gradually over the first 6 h, reaching a maximum at 10 h. To evaluate the reusability of the catalyst, BOC-3 was directly separated from the reaction system post-reaction through filtration or centrifugation, followed by washing and drying under vacuum. In five recycling experiments (Figure 4c), the catalyst exhibited a conversion rate of approximately 20%, indicating a degree of photostability. Subsequently, the BOC-3 catalyst was examined in the oxidation of other β-O-4 lignin model compounds, including 2-(2,6-dimethoxyphenol)-1-phenylethanone, 2-(2-methoxyphenoxy)-1-(4-methoxyphenyl)ethenone, 3-hydroxy-1-(4-hydroxy-3-methoxyphenyl)-2-(2-methoxyphenoxy)propan-1-one, and 1-(3,4-dimethoxy phenyl)-2-(2-methoxyphenoxy)-1,3-propanediol. In Table S2, BOC-3 effectively depolymerized the lignin model, achieving conversion rates ranging from 55% to 99% within 10 h, thereby demonstrating its excellent photocatalytic performance for complex lignin models under mild conditions.

To identify the active species involved in the reaction process, ESR spectroscopy was employed to detect •O_2_^−^ and •OH. Notably, the characteristic strong peaks corresponding to an •O_2_^−^ signal were observed under visible light irradiation in both BOC and BOC-3 materials [31] (Figure 4e). In Figure 4f, four signals indicative of •OH (in a 1:2:2:1 ratio) were detected under visible light in the BOC-3 sample [32]. According to the literature, it is plausible that •OH was synthesized through the reduction of •O_2_^−^: the reduction of O_2_ can first occur thanks to a photoelectron, followed by protonation to yield OOH. Subsequently, another reduction/protonation step converts OOH to H_2_O_2_, which is finally cleaved into •OH [33]. To further investigate the reaction mechanism associated with C-O bond cleavage, a series of control experiments were conducted. Initially, the photocatalytic effect was assessed under varying reaction conditions. The results in Table 1 (Entries 1 and 2) indicate that both light irradiation and the presence of a catalyst are essential for this reaction. Additionally, minimal product formation was observed under an N_2_ atmosphere (Table 1, Entry 3), resulting in a significantly reduced conversion rate of 34%. To elucidate the active species involved in the reaction, various scavengers were employed: thiobarbituric acid for •OH, K_2_S_2_O_8_ for photogenerated electrons, (NH_4_)_2_C_2_O_4_ for photogenerated holes, and *p*-benzoquinone for •O_2_^−^. The addition of K_2_S_2_O_8_ (Table 1, Entry 5) did not significantly suppress the conversion rate, although a slight reduction in product yield was noted. In contrast, (NH_4_)_2_C_2_O_4_ and *p*-benzoquinone exhibited a minor inhibitory effect on substrate conversion, resulting in a decrease of 20% (Table 1, Entries 6 and 7). The introduction of C_4_H_4_N_2_O_2_S inhibited both conversion (51%) and yield (Table 1, Entry 4), suggesting that •OH is the primary active species responsible for the depolymerization of the lignin model. Based on the aforementioned results, it can be concluded that the effect of active species on the reaction, ranked from highest to lowest, is outlined as •OH, h^+^, •O_2_^−^, and e^−^.

Due to the significant disparity between C-C and C-O bond cleavage, it has been estimated that the C_β_-O bond is cleaved first, resulting in the formation of 2-hydroxyacetophenone and phenolic compounds, as previously reported [34]. Concurrently, 2-hydroxyacetophenone and guaiacol (Figure S5c) were identified as products in the photocatalytic system through GC-MS. When 2-hydroxyacetophenone was utilized as a substrate, a conversion rate of 98% was achieved, yielding 89.46% selectivity for benzoic acid and 8.98% selectivity for benzaldehyde over BOC-3 catalyst after 10 h under the same conditions as previously described (Scheme S1, Equation (S2)). This result indicates that a portion of benzoic acid and benzaldehyde was not produced using direct cleavage of the C-C bond. To further investigate the pathways of the photocatalytic reaction, an isotope labeling experiment was conducted. In contrast to the depolymerization of 2-(2-methoxyphenoxy)-1-acetophenone, the aggregate production rate of aromatic monomers and the overall conversion decreased significantly in the isotope study. This phenomenon is attributed to the differences between H and D species [35]. According to the results obtained from GC-MS analysis, C_β_-D exhibits a different signal at *m*/*z* = 138 in phCOCD_2_OH (Figure 4c), which confirms that 2-hydroxyacetophenone originates from C-O bond cleavage in 2-(2-methoxyphenoxy)-1-acetophenone. Additionally, signals at *m*/*z* = 106 and 120 were detected (Figure S5b,d), suggesting that benzaldehyde and acetophenone are generated under the reaction conditions.

**Figure 5:**
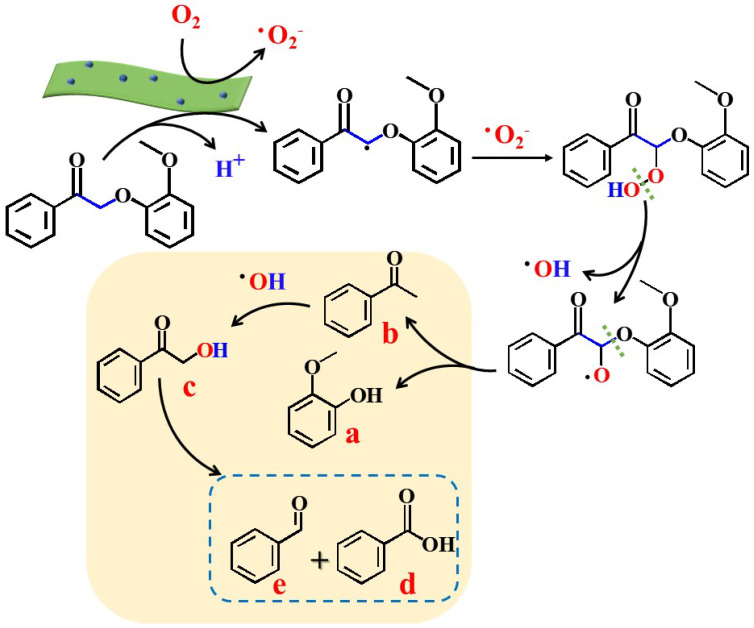
Proposed reaction pathways for photocatalytic conversion of 2-(2-methoxyphenoxy)-1-phenylethanone catalyzed by BOC-3.

Based on the aforementioned results, this study proposes a feasible reaction mechanism that involves H abstraction at the β position (Figure 5). The incorporation of S was achieved by substituting Cl atoms in BOC [17], which generates impurity levels and defects within BOC layers. The synergistic effect of these two elements not only increases the number of adsorption sites and the reduction capacity of O_2_ but also enhances the separation and migration of photogenerated charge carriers [36]. The absorbed O_2_ is reduced to •O_2_^−^ by photogenerated electrons. Subsequently, photogenerated holes activate the C_β_-H bond, leading to the C_β_-O• radical (which further forms a C_β_ = O ketone intermediate). •O_2_^−^ species then react with these unstable intermediates to produce peroxide intermediates. Ultimately, lignin facilitates the cleavage of the C-O bond, resulting in the formation of guaiacol, acetophenone, and 2-hydroxyacetophenone. Furthermore, 2-hydroxyacetophenone is unstable within the reaction system and can undergo further depolymerization into phenol, benzoic acid, benzaldehyde, and acetophenone, achieving a conversion rate of 98%.

## 3. Methods

### 3.1. S/BOC Preparation

**Synthesis of ultrathin BOC nanosheet.** BOC nanosheets were conducted in accordance with methods in Xu et al. [37]. Firstly, 3.88 g of Bi(NO_3_)_3_•5H_2_O (Search banner reagent (Shanghai) Co., Ltd., Shanghai, China) and 0.234 g of NaCl (Sinopharm Chemical Reagent Co., Ltd., Shanghai, China) were dissolved in 48 mL of deionized water and stirred at room temperature until fully dissolved. Secondly, the pH of the solution was adjusted to 11 using 25 wt.% NH_3_ aqueous solution (Sinopharm Chemical Reagent Co., Ltd., Shanghai, China), and stirring was continued for an additional 30 min. The resulting solution was then transferred to a high-pressure reactor equipped with a 100 mL polytetrafluoroethylene lining, where the reaction was conducted at 220 °C for 18 h. After cooling to room temperature, the product was collected, rinsed three times with deionized water and absolute ethanol, and then dried in a vacuum at 50 °C.

**Synthesis of S-doped ultrathin BOC nanosheet.** S-doped BOC nanosheets were synthesized using a simple hydrothermal method. Specifically, thiourea (Search banner reagent (Shanghai) Co., Ltd., Shanghai, China) of different masses (1.1 mg, 3.1 mg, 5.1 mg, and 7.1 mg) were combined with 0.1 g of BOC in a total volume of 20 mL of deionized water under magnetic stirring. After 10 min of stirring, the resulting solution was transferred to a high-pressure reaction vessel lined with 50 mL of polytetrafluoroethylene and maintained at a temperature of 180 °C for 6 h. Following the reaction, the mixture was allowed to cool to room temperature, after which the product was collected, washed three times with deionized water, and dried in a vacuum at 70 °C. S content in BOC nanosheets was quantified using inductively coupled plasma (ICP) analysis (Table S1). The weight percentages of S in BOC nanosheets were determined to be 1.0%, 1.5%, 2.0%, and 2.5%, corresponding to the samples designated as BOC-1, BOC-2, BOC-3, and BOC-4, respectively.

### 3.2. Photocatalytic Lignin Depolymerization

The photocatalytic depolymerization of lignin was conducted in a 10 mL quartz reactor. A solution containing 0.05 mmol of 2-(2-methoxyphenoxy)-1-phenylethanone and 5 mg of the catalyst was prepared in 1 mL of acetonitrile. The reactor was then purged with O_2_ to create an appropriate atmosphere for the reaction, which was carried out for 30 min under dark conditions. A 300 W Xenon lamp, equipped with an AM 1.5G optical filter, served as the light source positioned alongside the reactor. During the irradiation process, magnetic stirring was maintained, and the reaction temperature was regulated to room temperature using an electric fan (Supplementary Materials). Following the reaction, the solution was filtered through a 0.22 μm filter and subsequently analyzed using gas chromatography (GC, Agilent 7820A, Santa Clara, CA, USA). The depolymerization of deuterated lignin models was further examined by gas chromatography–mass spectrometry (GC-MS-CLARUS500, PerkinElmer, Cambridge, MA, USA), with detailed calculation results documented in the Supplementary Materials.

## 4. Conclusions

This study synthesized a series of S-doped BOC-3 photocatalysts using a simple hydrothermal method, which were investigated for their capacity to cleave C-O bonds in lignin models. The results demonstrate that BOC-3 nanosheets effectively depolymerize C-O bonds, yielding aromatic compounds under visible light at room temperature. Both theoretical and experimental analyses confirm that S doping enhances photogenerated carrier migration and separation while also increasing O adsorption and reduction sites. These improvements lead to an elevated concentration of •O_2_^−^ radicals, which, in combination with peroxide intermediates, facilitate efficient C-O bond cleavage. This research highlights the potential of visible-light-driven catalyst interface engineering as a promising approach for lignin depolymerization, establishing a foundation for the development of efficient photocatalysts in sustainable biomass conversion.

## Figures and Tables

**Table 1:** Product distribution and conversion efficiency under various reaction conditions for photocatalytic C-O bond cleavage in lignin β-O-4 model.

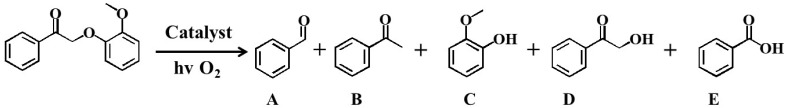									
Entry	BOC-3	hv	O_2_	Conversion	Yield (µmol/g/h)				
				(%)	A	B	C	D	E
1	**−**	**+**	**+**	43.99	0.08	18.00	0.99	0.16	1.24
2	**+**	**−**	**+**	13.29	4.06	2.03	2.03	8.12	9.14
3 ^a^	**+**	**+**	**−**	34.21	4.00	7.44	33.26	8.00	51.46
4	**+**	**+**	**+**	99.43	11.29	7.72	182.85	20.36	406.22
5 ^b^	**+**	**+**	**+**	37.57	7.62	775	36.61	21.87	57.87
6 ^c^	**+**	**+**	**+**	83.41	4.00	5.09	2.00	61.82	155.32
7 ^d^	**+**	**+**	**+**	67.36	3.98	8.17	18.79	50.79	130.37
8 ^e^	**+**	**+**	**+**	61.46	4.21	5.86	95.54	80.63	176.46
9 ^f^	**+**	**+**	**+**	60.9	3.86	10.15	225.47	13.31	88.44

Reaction conditions, unless otherwise specified: 0.05 mmol 2-(2-methoxyphenoxy)-1-phenylethanone, 10 mg of BOC-3 catalyst, 2 mL CH_3_CN, under an O_2_ atmosphere, with Xe lamp (300 W) for 10 h. ^a^ Reaction under N_2_ atmosphere. ^b^ Addition of 0.05 mmol C_4_H_4_N_2_O_2_S. ^c^ Addition of 0.05 mmol K_2_S_2_O_8_. ^d^ Addition of 0.05mmol (NH_4_)_2_C_2_O_4_. ^e^ Addition of 0.05 mmol *p*-benzochinone. ^f^ Addition of 0.05 mmol DMPO.

## Data Availability

Data are contained within the article and Supplementary Materials.

## Supplementary Materials

The following supporting information can be downloaded at https://www.mdpi.com/article/10.3390/molecules29245979/s1, Catalyst characterization device; DFT calculations; lignin model compound synthesis step; NMR data and spectra of lignin model compounds; ICP test over S/Bi3O4Cl with different S doping; schematic of the synthesis process of BOC-3; SEM images of BOC; AFM images of BOC-3 and the corresponding height profiles of lines; Roman shift of BOC and BOC-3 samples; XPS survey spectra of BOC and BOC-3 samples; UV–vis DRS spectra and the plots of (ahν)^1/2^ vs. photon energy (hν) of BOC and BOC-3; Mott–Schottky plots of BOC and BOC-3; M–S analyses of deuterated 2-(2-methoxyphenoxy)-1-acetophenone, PhCOH, PhOCH_3_OH, PhCOCH_3_; energy band structure of BOC and BOC-3. References [38,39,40,41] are cited in the Supplementary Materials.
