# Supplementary material for: Photocatalytic Depolymerization of Lignin: C-O Bond Cleavage in β-O-4 Models Using S-Doped Ultra-Thin Bi3O4Cl Nanosheets
Source: Molecules. 2024 Dec 18;29(24):5979. doi: 10.3390/molecules29245979 (PMC11679700; doi:10.3390/molecules29245979)
Supplement: Supplementary file 1 [file molecules-29-05979-s001.zip › molecules-3311568-supplementary.pdf]

Supplementary Materials

Photocatalytic depolymerization of lignin: C–O bond cleavage in β-O-4 models using S-doped ultra-thin Bi_3O_4Cl nanosheets

Chunli jiang [a,b,c*], Sixue Zhang[b]

[a] School of Chemistry, Sun Yat-Sen University, Guangzhou 510275, P.R., China.

[b] School of Chemistry and Chemical Engineering, Guangxi Minzu University, Nanning 530006, China.

[c] Hui Zhou Research Institute, Sun Yat-Sen University, Huizhou 516000, P.R., China.

1.Experomental section

1.1 Lignin model compounds Synthesis.

1) Procedure for preparation of 2-(2-methoxyphenoxy)-1-phenylethanone

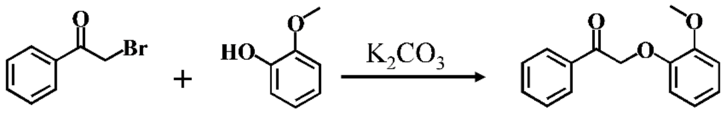

2-bromoacetophenone (7.36g, 36.9 mmol). 2-methoxy-phenol (5.74g,46.24 mmol) and K_2CO_3 (9.2g, 66.57mmol) were added sequentially to 150ml acetone and condensed and refluxed in an oil bath at 60°C for 12h. After the reaction, the potassium carbonate was washed 3 times with ether, the organic phases were combined and the solvent was evaporated by rotation. The solid residue was washed 3 times with ether to remove unreacted α-bromoacetophenone and guaiacol, and dried under vacuum at 45°C to obtain white crystals[1].

2) Procedure for preparation of 2-(2,6-dimethoxyphenoxy)-1-phenylethanone

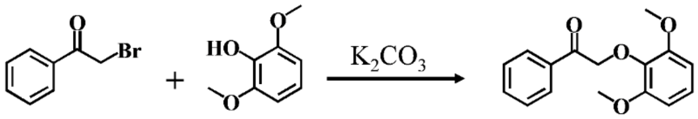

2-bromoacetophenone (2.5 g 15.7 mmol), 2.6-dimethoxyphenol (2.5 g, 12.6 mmol) and K2CO3 (12.3 g, 89.1 mmol) were dispersed in 250 ml acetone, the mixture refluxed at 70℃ for 12 h. After the reaction, the reactants are cooled to room temperature, the suspension is filtered and the filtrate is concentrated under vacuum, and the precipitated solid is recrystallized in anhydrous ethanol three times to obtain a white solid[2-3].

3) Procedure for preparation of 2-(2-Methoxyphenoxy)-1-(4-methoxyphenyl) ethenone

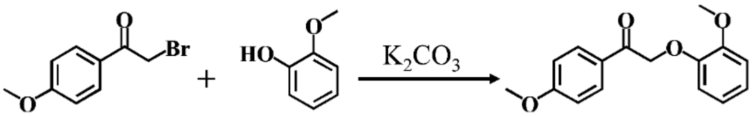

α-bromo-4-methoxyacetophenone (2.8 g 12mmol), 2-methoxy-phenol (1.6 g, 13.2mmol) and K_2CO_3 (2.5 g, 18mmol) were dispersed in 50 ml acetone, the mixture refluxed at 30℃ for 12 h under N_2 atmosphere. After the reaction, the suspension is

filtered and the filtrate is concentrated under vacuum, the obtained oily product is dissolved in warm ethanol and cooled to 0° for recrystallization. Finally, the crystallized solids are dried under vacuum at 45°C to obtain white crystals[1].

4）Procedure for preparation of 3-hydroxy-2-(2-methoxyphenoxy)-1-(4-methoxyphenyl) propan-1-one

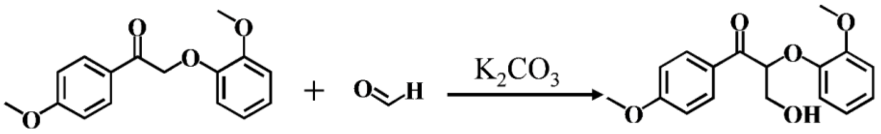

2-(2-Methoxyphenoxy)-1-(4-methoxyphenyl) ethenone (0.92 g 3.4mmol), K_2CO_3 (0.51g, 3.7mmol) were dissolved in 20ml acetone/ethanol (1:1), stirring the mixture at room temperature for 5min. Then adding formalin (0.15g 3.1mmol) and stir for another 2 hours. After the reaction, the suspension is filtered and vacuum concentrated. The as-prepared oily substance was extracted three times by organic solvent, then the organic phase was dried with $MgSO_4$, filtered and concentrated. Finally, the crystallized solids are dried under vacuum at 60°C to obtain light solid.

5）Procedure for synthesis of the deuterated compounds

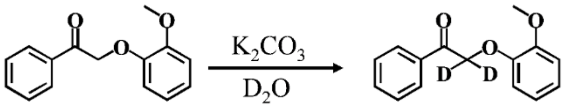

Deuterated lignin model compound according to the method in the literature[2]. 2-(2-methoxyphenoxy)-1-phenylethanone (0.50g,2.06mmol) was added to a sealed bottle with anhydrous K_2CO_3 (0.126g,0.92mmol) and 5ml of D_2O. The reaction ran at 100 °C for 24h. After the reaction, sucked out the heavy water and replaced it by fresh one. Then reaction was maintained for additional 24h at 100 °C, then wash the reaction solid to remove K_2CO_3 residues. Finally, the solid was dried under vacuum to give deuterated compounds as a light yellow solid in 95% yield[4].

1.2 Catalyst Characterization

Crystal structures were analyzed using powder X-ray diffraction (XRD) with a Rigaku MiniFlex600 instrument employing Cu Kα radiation (λ=0.15406 nm) over a 2θ range of 10° to 80°. The micromorphology of the samples was characterized through

scanning electron microscopy (SEM) utilizing a Carl Zeiss instrument. Microstructural observations were conducted using a transmission electron microscope (TEM, JEM-2100Plus, JEOL) at an accelerating voltage of 200 kV, complemented by high-resolution transmission electron microscopy (HRTEM). Atomic force microscopy (AFM) analyses were performed with a MultiMode8 instrument. X-ray photoelectron spectroscopy (XPS) measurements were executed using a Thermo Scientific Nexsa instrument, which is equipped with a monochromatic Al Kα radiation source. Ultraviolet-visible (UV–Vis) absorption spectra of the photocatalysts were obtained using diffuse reflectance spectroscopy (DRS, Lambda 1050+) within the wavelength range of 200~800 nm, employing $BaSO_4$ as a reference material. Photocurrent measurements and electrochemical impedance spectroscopy (EIS) were conducted using a standard three-electrode electrochemical workstation (CS312M) with a 0.1 M Na_2SO_4 electrolyte solution. The electrodes utilized included a Pt sheet as the counter electrode, an Ag/AgCl electrode as the reference, and an indium tin oxide (ITO) substrate coated with the catalyst as the working electrode. EIS was performed across a frequency range of 0.1~10^5 Hz, while Mott-Schottky plots were generated at a frequency of 1500 Hz. Steady-state photoluminescence (PL) spectra and time-resolved photoluminescence (TRPL) decay measurements were conducted using an FLS-1000 spectrometer, with an excitation wavelength of 365 nm. The attenuation curves were fitted using a double exponential attenuation function, and the average PL lifetimes were calculated according to the following equation:

$$A(t)=A_1 e^{(-\frac{t}{\tau_1})}+A_2 e^{(-\frac{t}{\tau_2})}$$

$$\tau_{average}=(A_1\tau_1^2+A_2\tau_2^2)/(A_1\tau_1+A_2\tau_2)$$

1.3 DFT Calculations.

All calculations are conducted within the framework of density functional theory (DFT) utilizing projector augmented wave (PAW) method, as implemented in the Vienna ab initio simulation package (VASP). Generalized gradient approximation (GGA) proposed by Perdew, Burke, and Ernzerhof is employed for the exchange-

correlation potential. The van der Waals interactions are accounted for using DFT-D3 approach. A cutoff energy of 450 eV is established for the plane wave basis set. The energy convergence criterion is set to 10^{-5} eV during the iterative solution of the Kohn-Sham equations. To prevent artificial interactions between periodic images, a vacuum layer of 15 Å is introduced perpendicular to the sheet. Brillouin zone integration is performed using a 2×2×1 k-mesh with Monkhorst-Pack sampling scheme[4]. All structures are optimized until the residual forces acting on the atoms are reduced to less than 0.02 eV/Å. The adsorption energy E_{ad} is defined as follows:

$$E_{ad}=E_{all}-E_{subs}-E_{o_2}$$

where E_{all}, E_{subs} and E_{O2} represent the energy of the total system, the energy of the substrate, and that of an isolated O_2 molecule, respectively.

1.4 photo of the reactor

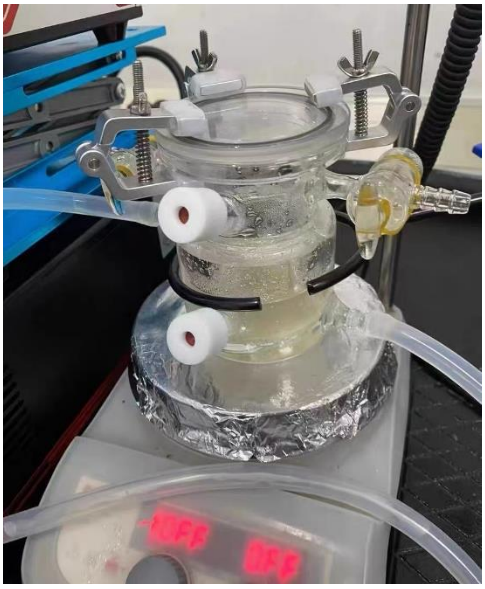

During the photocatalytic process, the reaction system was vigorously stirred with a magnetic stirrer, the cooling water system was maintained at 30℃.

2. Supplementary Results

NMR data and spectra of lignin model compounds.

1）2-(2-methoxyphenoxy)-1-phenylethanone

^{1}H NMR (400 MHz, $CDCl_3$) δ = 8.02 – 8.0 (m, 2H), 7.60 (t, J=7.4 Hz, 1H), 7.51 (t, J=7.7 Hz, 2H), 7.02 – 6.82 (m, 4H), 5.35 (s, 2H), 3.88 (s, 3H). 13C NMR (101 MHz, $CDCl_3$) δ = 194.89, 147.60, 134.73, 133.79, 128.82, 128.11, 122.52, 120.82, 114.91, 112.23, 72.11, 55.93. 133.86, 128.82, 128.19, 122.59, 120.89, 114.99, 112.31, 72.20, 56.01.

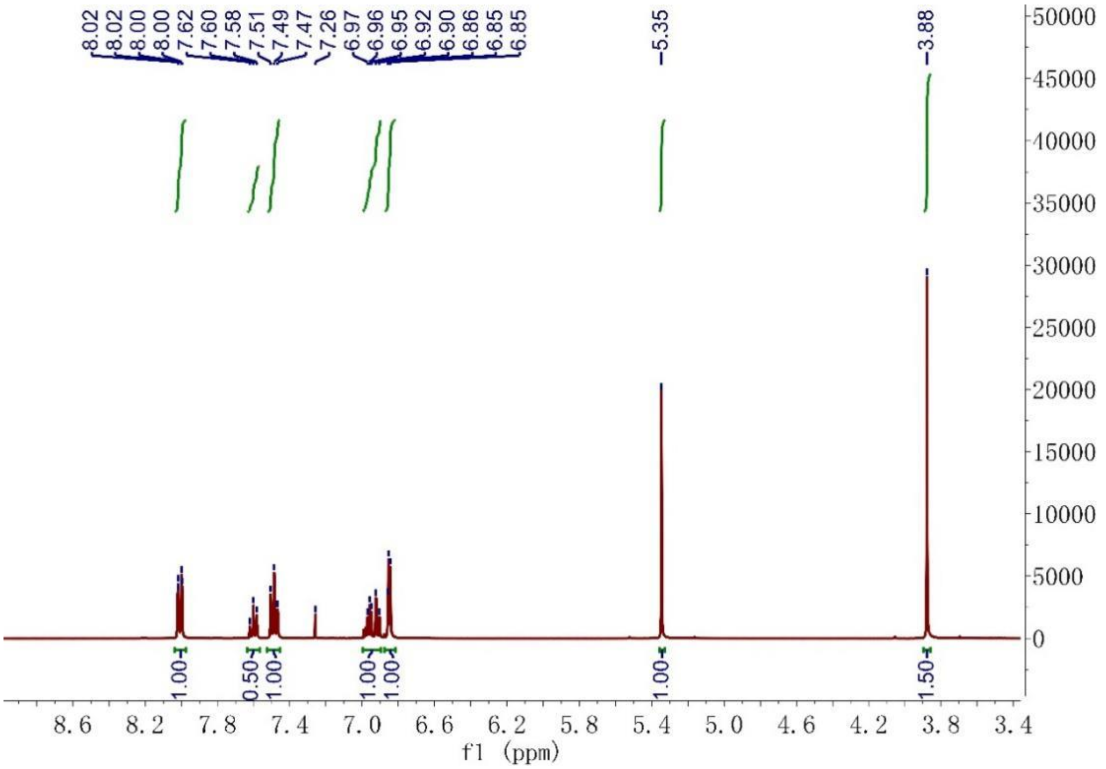

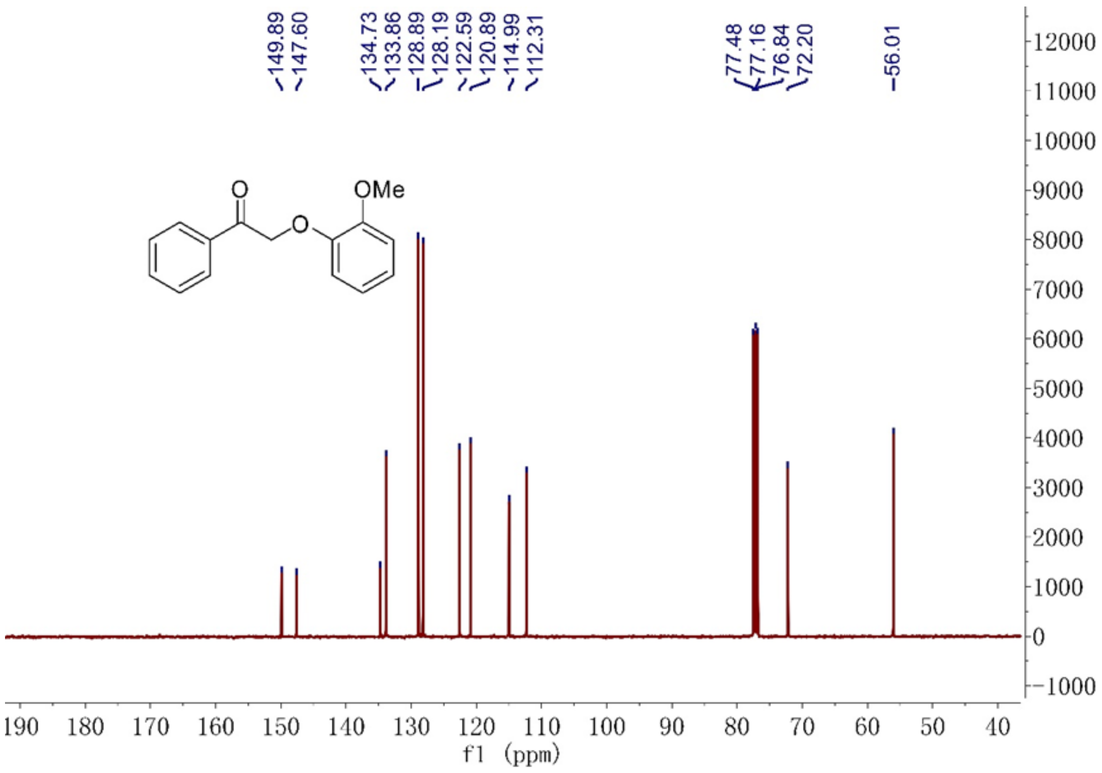

2) 2-(2,6-dimethoxyphenoxy)-1-phenylethanone

^{1}H NMR (400 MHz, $CDCl_3$) δ = 8.05 (d, *J* = 7.7 Hz, 2H), 7.59(t, *J* = 7.3 Hz, 1H),7.46 (t, *J* = 7.6 Hz, 2H), 7.00 (t, *J* = 8.4 Hz, 1H), 6.58 (d, *J* = 8.4 Hz, 2H), 5.20 (s,2H), 3.80 (s, 6H). ^{13}C NMR (101 MHz, $CDCl_3$) δ =195.29, 153.26, 136.73, 135.27,133.37, 128.64, 128.41, 124.17, 105.42, 75.48, 56.16.

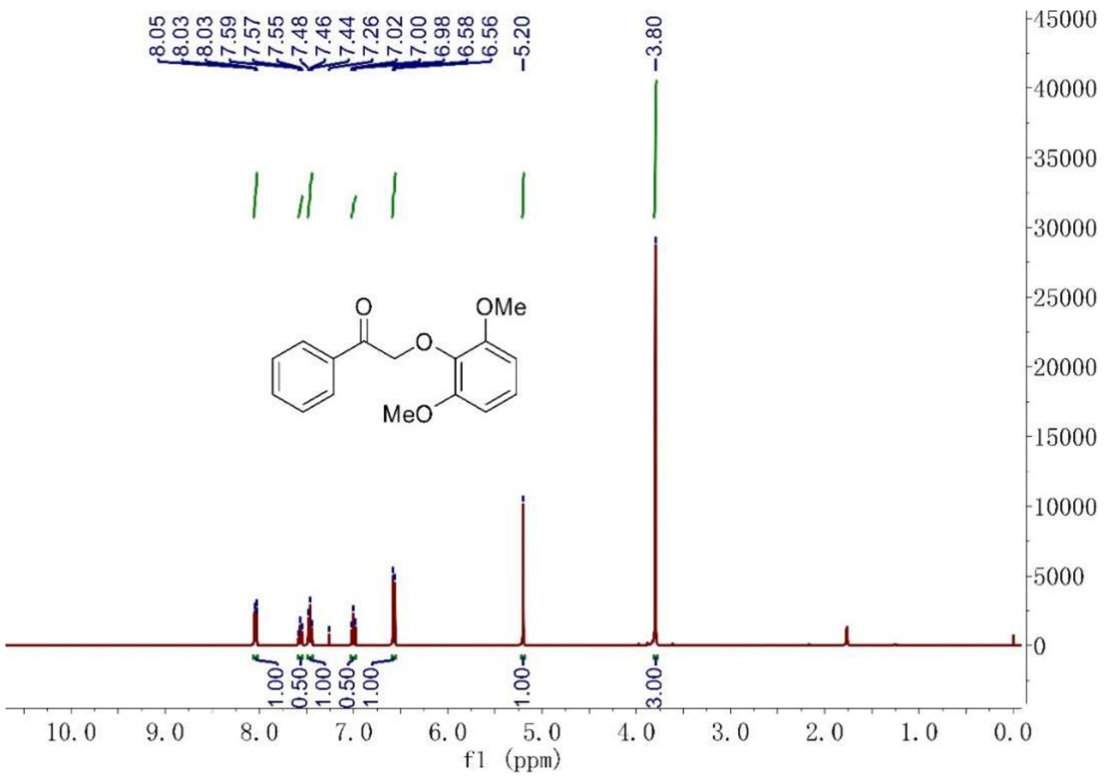

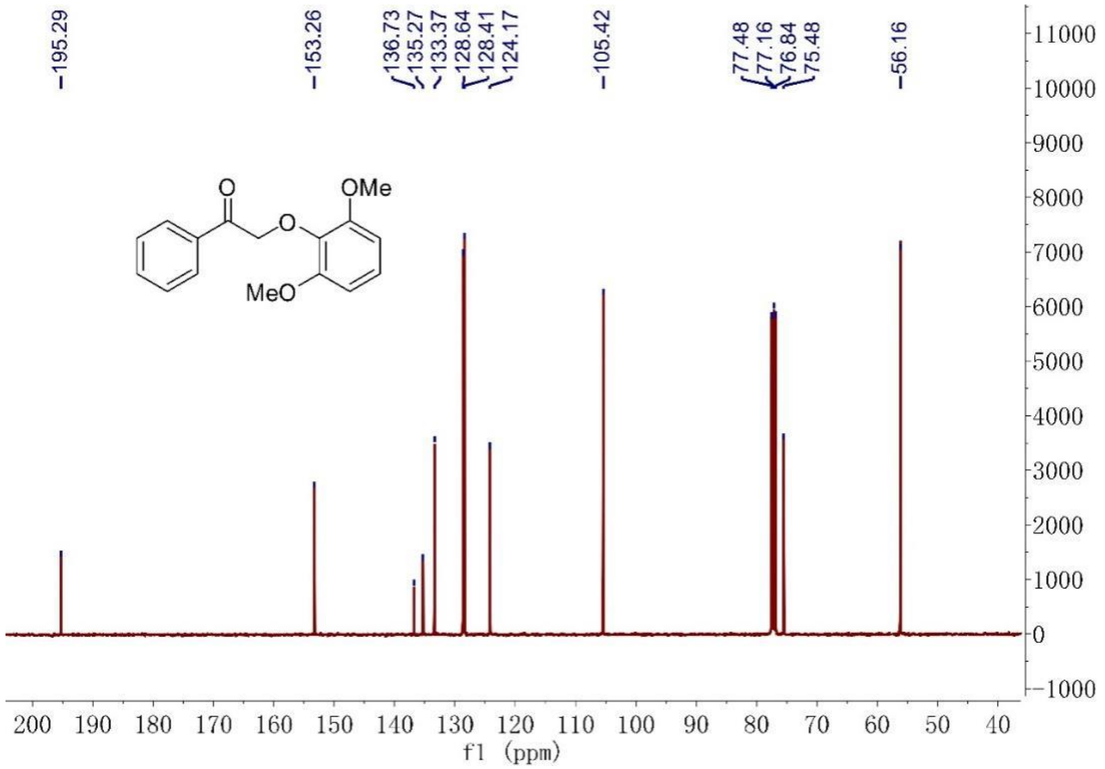

3) 2-(2-Methoxyphenoxy)-1-(4-methoxyphenyl) ethenone

^{1}H NMR (400 MHz, $CDCl_3$) δ=8.02 (d, J = 8.8 Hz, 2H), 7.10-7.07 (m, 2H), 6.98(d, J = 7.5 Hz, 2H), 6.3-6.88 (m, 2H), 5.33 (s, 2H), 3.86 (s, 3H), 3.78-3.35 (m, 3H). ^{13}C NMR (101 MHz, $CDCl_3$) δ=193.52, 164.01, 149.43, 147.97, 130.74, 127.83, 121.76,

121.00, 114.51, 114.09, 112.91, 70.94, 56.09, 40.60, 39.98.

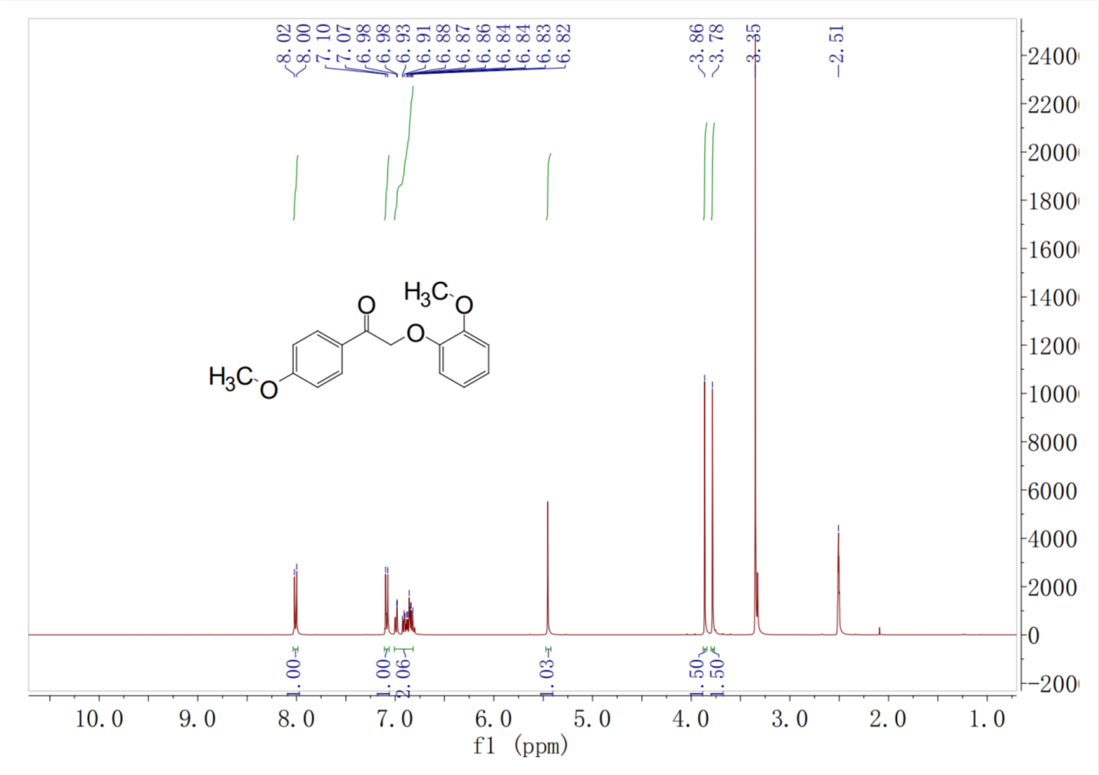

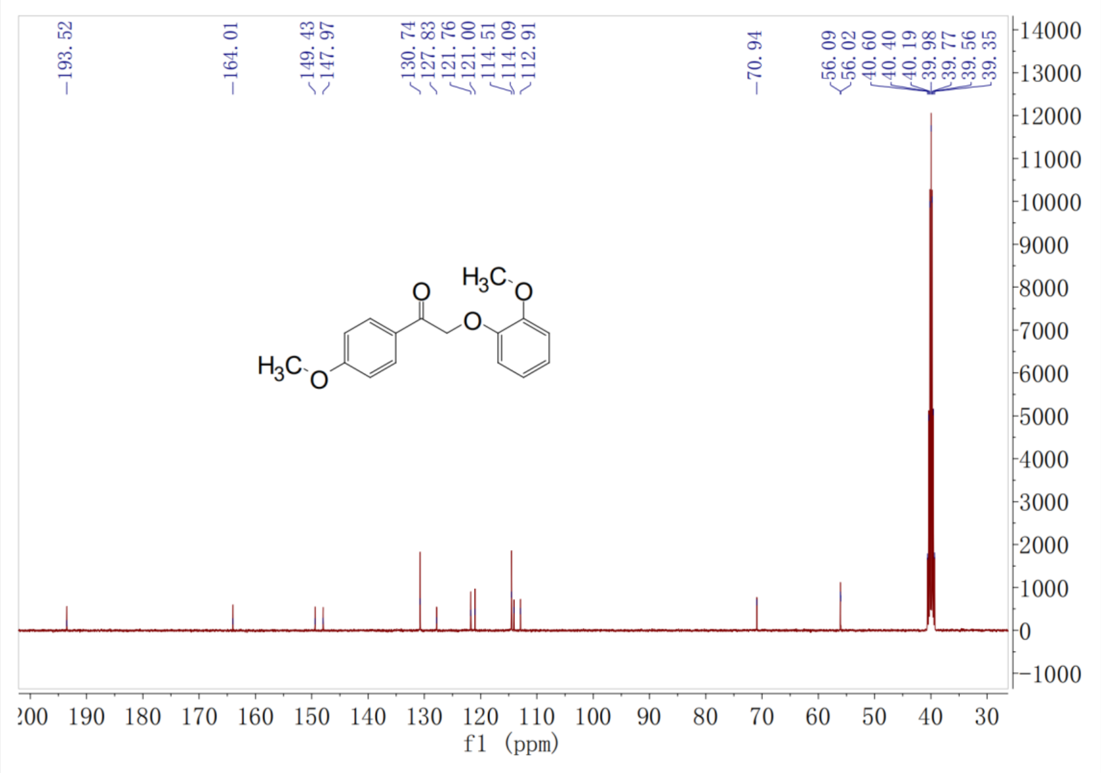

4）3-hydroxy-2-(2-methoxyphenoxy)-1-(4-methoxyphenyl) propan-1-one

^{1}H NMR (400 MHz, $CDCl_3$) δ 8.07-8.05 (m, 2H), 6.93-6.91 (m, 1H), 6.87 (ddt, J = 11.7, 8.0, 2.3 Hz, 4H), 6.91-6.79 (m, 1H), 5.2 (t, J = 5.2 Hz, 1H), 4.07 (d, J = 5.2 Hz,

2H), 3.82 (s, 3H), 3.90 (s, 3H), 2.82 (s, 1H). ^{13}C NMR (101 MHz, $CDCl_3$) δ 195.07, 164.06, 150.13, 146.52, 131.25, 127.05, 123.25, 121.13, 117.59, 114.00, 112.27, 63.58, 55.80, 55.54.

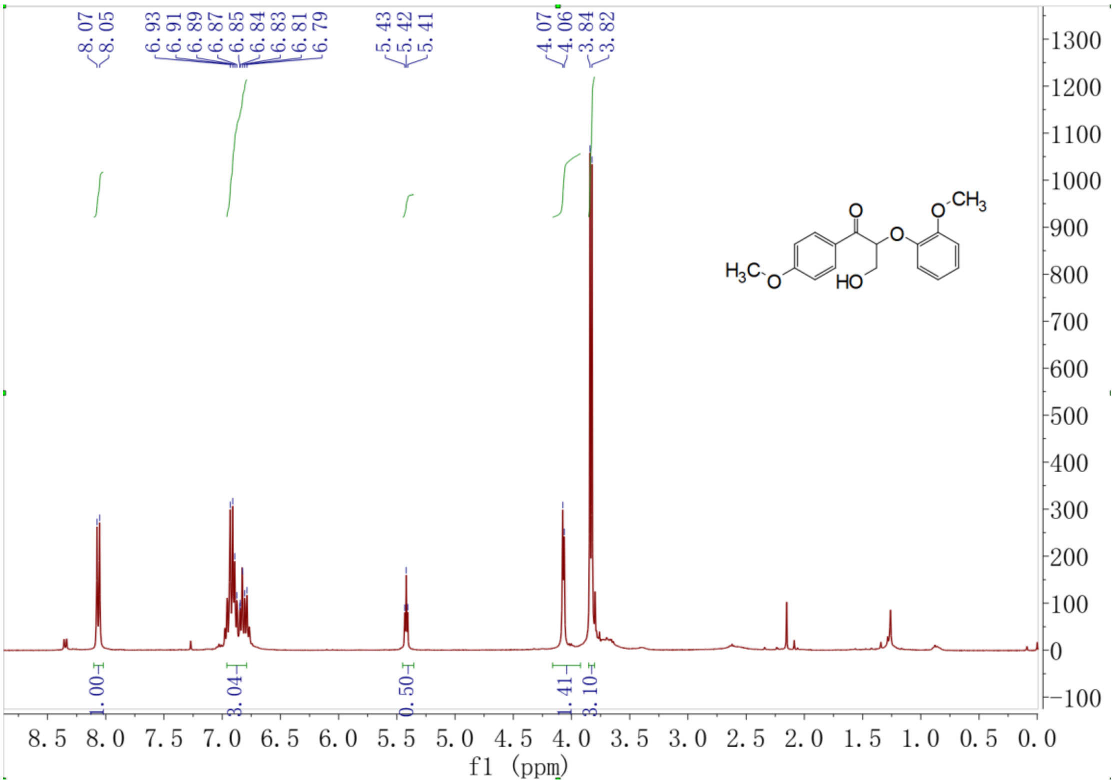

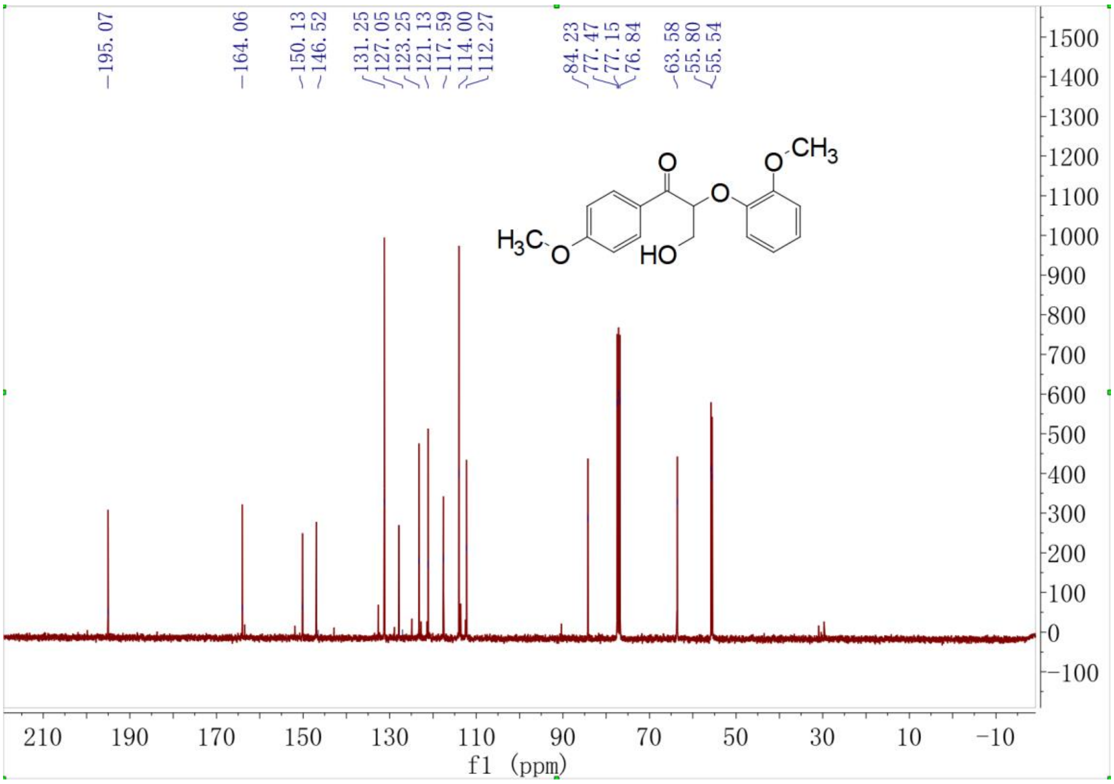

Other data

Yield of product and selectivity for product were calculated with following equations:

$$Conversion(\%) = \frac{moles\ substrate\ input - moles\ of\ substrate\ output}{moles\ of\ substrate\ input} \times 100\%$$

$$Yield(umol/g(Ct)/h = \frac{moles\ of\ corresponding\ product}{mass\ of\ catalyst \times rection\ time}$$

$$Selectivity(\%) = \frac{moles\ of\ (c+d+e)}{moles\ of\ (a+b+c+d+e)} \times 100\%$$

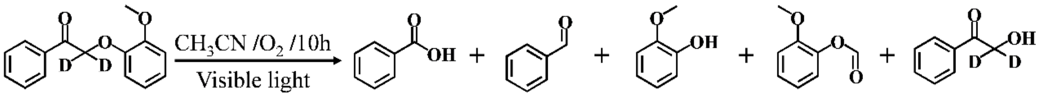

(S1)

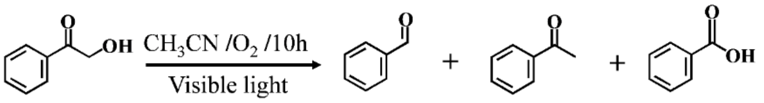

(S2)

Scheme S1 Controlled reaction equation

Table S1.ICP test over S/ Bi_3O_4Cl with different S doping

number of samples	sample quality (mg)	S content detected by icp (ppm)	S content detected by icp(mg)	weight percentage (wt%)
1	20.7	21.09	0.21	1.02
2	21.7	34.91	0.35	1.61
3	20.9	43.71	0.44	2.09
4	20.4	57.95	0.58	2.84

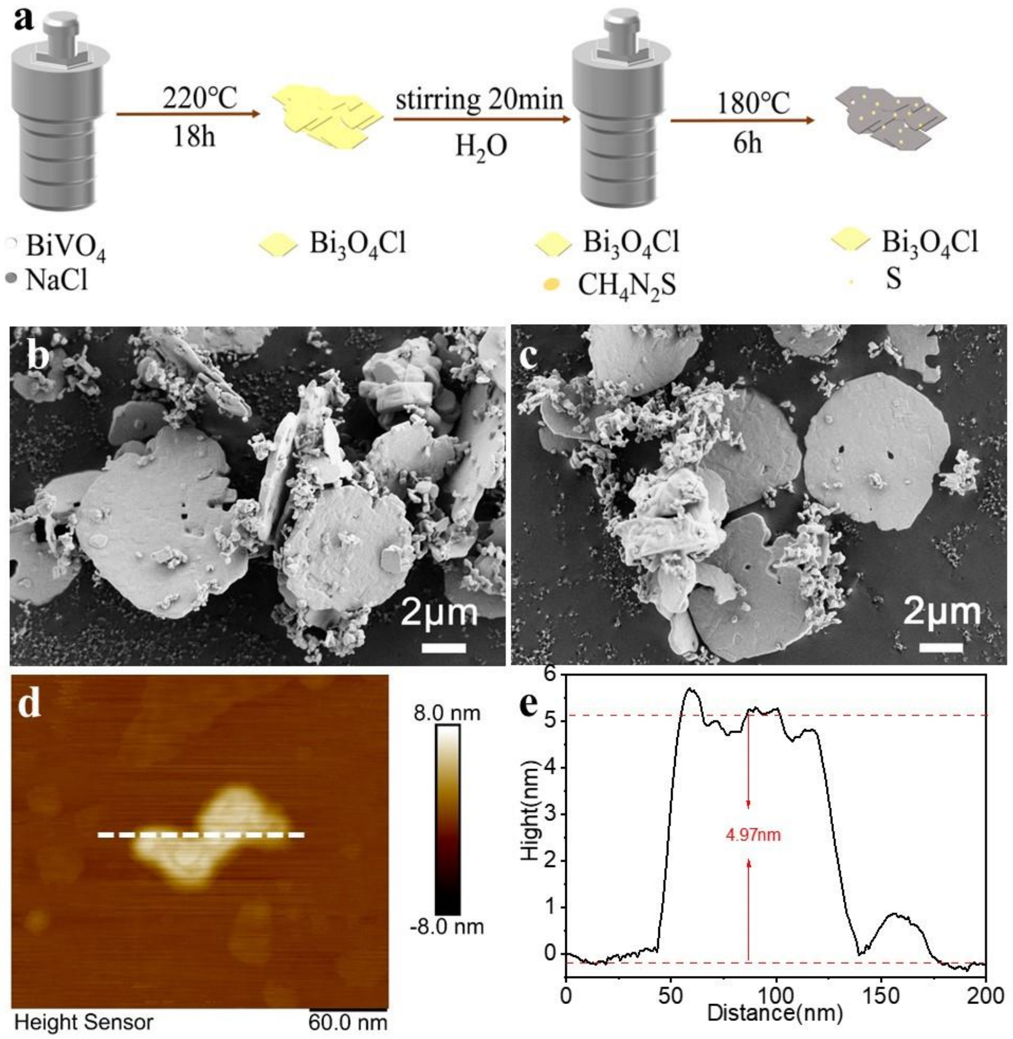

Figure S1 (a) Schematic of the synthesis process of BOC-3; (b-c) SEM images of BOC; (d) AFM images of BOC-3 and (e) the corresponding height profiles of lines

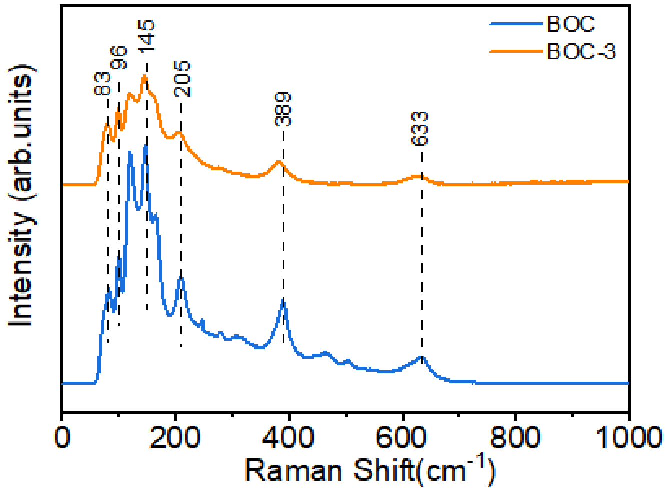

Figure S2 Raman shift of BOC and BOC-3 samples

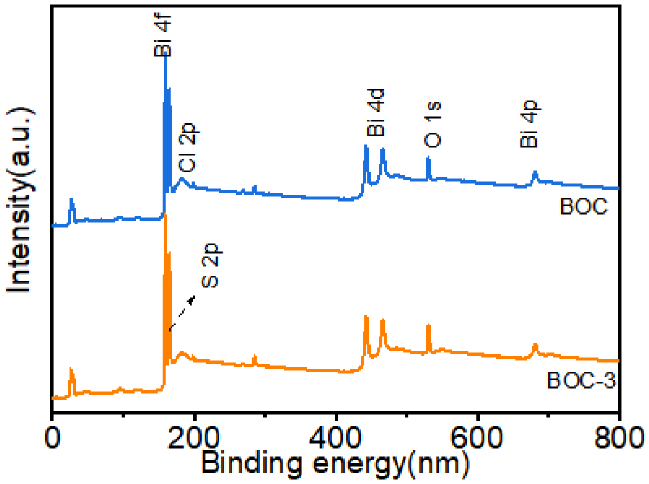

Figure S3 XPS survey spectra of BOC and BOC-3 samples

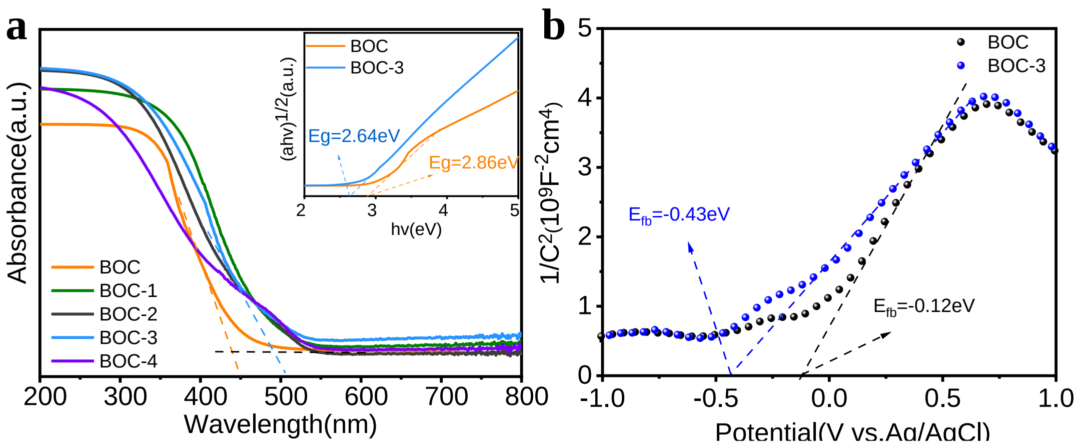

Figure S4 (a) UV–vis DRS spectra and the plots of $(ah\nu)^{1/2}$ vs. photon energy ($h\nu$) of BOC and BOC-3; (b) Mott-Schottky plots of BOC and BOC-3.

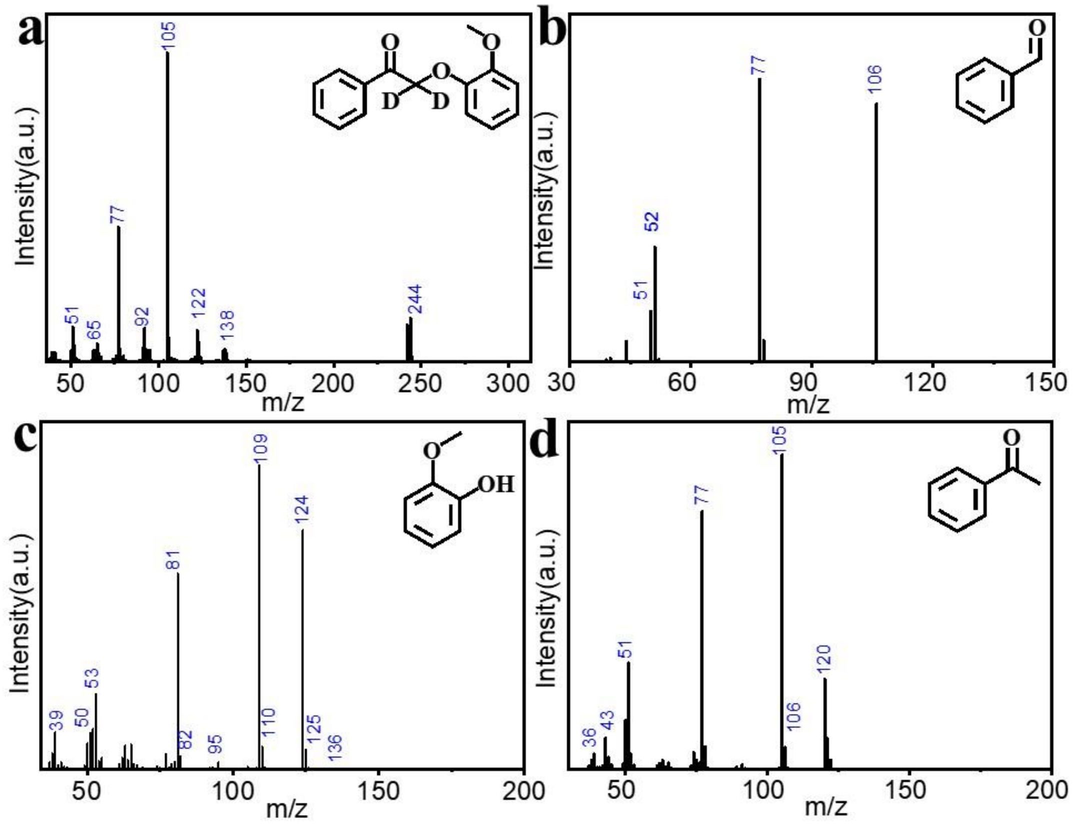

Figure S5 GC-MS analysis results of (a) deuterated 2-(2-methoxyphenoxy)-1-acetophenone; (b) PhCOH (c) $PhOCH_3OH$, (d) $PhCOCH_3$

Table S2. Photocatalytic conversion of different β-O-4 lignin model compounds with the BOC-3 photocatalyst under solar irradiation

Substrate	Time (h)	Conv. (%)	Main Products(Yield, μmol · g^{-1} · h^{-1})		
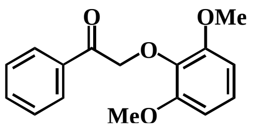	10	99.88	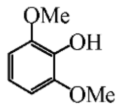 40.32	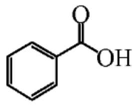 188.34	
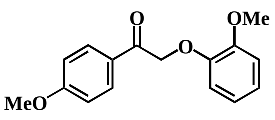	10	92.06	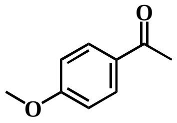 16.48	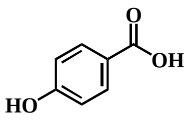 305.43	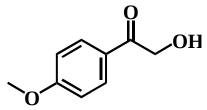 123.98
			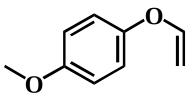 23.53		

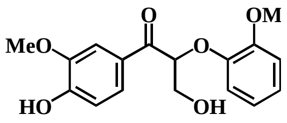	10	52.61	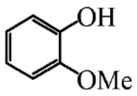 7.72	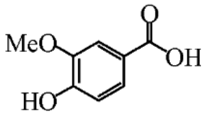 12.40	
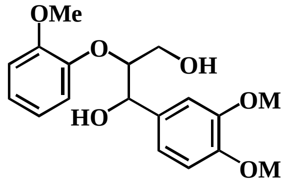	10	86.06	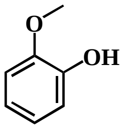 61.75	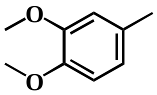 40.83	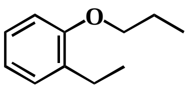 43.60
			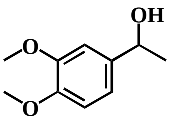 218.25	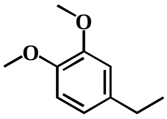 8.07	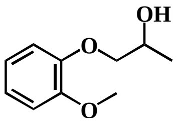 4.14

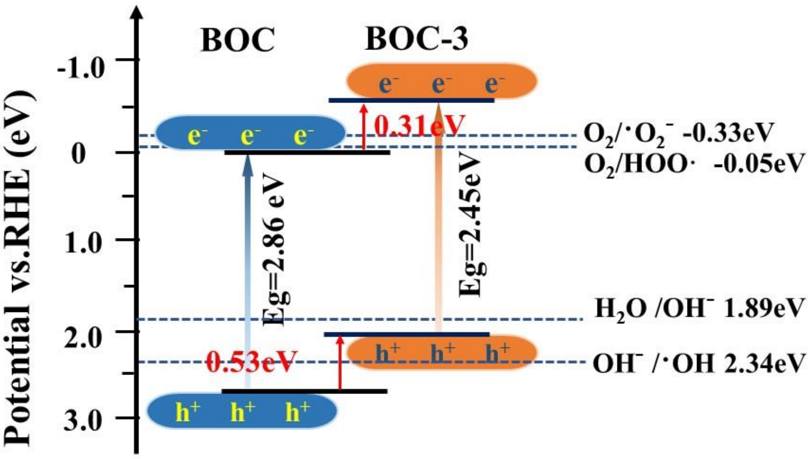

Figure S7 Energy band structure of BOC and BOC-3

3. References

[1] Rinesch T, Bolm C. Cobalt-Catalyzed Oxidation of the beta-O-4 Bond in Lignin and Lignin Model Compounds[J]. ACS Omega, 2018, 3(7): 8386-8392.

[2] Dawange M, Galkin M V, Samec J S M. Selective Aerobic Benzylic Alcohol Oxidation of Lignin Model Compounds: Route to Aryl Ketones[J]. ChemCatChem,2015,7(3): 401-404.

[3] Liu H, Li H, Lu J, et al. Photocatalytic Cleavage of C–C Bond in Lignin Models under Visible Light on Mesoporous Graphitic Carbon Nitride through π–π Stacking Interaction[J]. ACS Catalysis, 2018, 8(6): 4761-4771.

[4] Wang X, Chu S, Shao J, et al. Efficient and Selective C–C Bond Cleavage of a Lignin Model Using a Polyimide Photocatalyst with High Photooxidation Capability[J]. ACS Sustainable Chemistry & Engineering, 2022, 10(35): 11555-11566.
